# The use of artificial intelligence in musculoskeletal ultrasound: a systematic review of the literature

**DOI:** 10.1007/s11547-024-01856-1

**Published:** 2024-07-13

**Authors:** Jonas M. Getzmann, Giulia Zantonelli, Carmelo Messina, Domenico Albano, Francesca Serpi, Salvatore Gitto, Luca Maria Sconfienza

**Affiliations:** 1 IRCCS Istituto Ortopedico Galeazzi, Milan, Italy; 2 Dipartimento Di Scienze Biomediche Per La Salute, Università Degli Studi Di Milano, Milan, Italy; 3 UOC Radiodiagnostica, ASST Centro Specialistico Ortopedico Traumatologico Gaetano Pini-CTO, Milan, Italy; 4 Dipartimento Di Scienze Biomediche, Chirurgiche Ed Odontoiatriche, Università Degli Studi Di Milano, Milan, Italy

**Keywords:** Artificial intelligence, Machine learning, Deep learning, Musculoskeletal, Ultrasound

## Abstract

**Purpose:**

To systematically review the use of artificial intelligence (AI) in musculoskeletal (MSK) ultrasound (US) with an emphasis on AI algorithm categories and validation strategies.

**Material and Methods:**

An electronic literature search was conducted for articles published up to January 2024. Inclusion criteria were the use of AI in MSK US, involvement of humans, English language, and ethics committee approval.

**Results:**

Out of 269 identified papers, 16 studies published between 2020 and 2023 were included. The research was aimed at predicting diagnosis and/or segmentation in a total of 11 (69%) out of 16 studies. A total of 11 (69%) studies used deep learning (DL)-based algorithms, three (19%) studies employed conventional machine learning (ML)-based algorithms, and two (12%) studies employed both conventional ML- and DL-based algorithms. Six (38%) studies used cross-validation techniques with K-fold cross-validation being the most frequently employed (*n* = 4, 25%). Clinical validation with separate internal test datasets was reported in nine (56%) papers. No external clinical validation was reported.

**Conclusion:**

AI is a topic of increasing interest in MSK US research. In future studies, attention should be paid to the use of validation strategies, particularly regarding independent clinical validation performed on external datasets.

## Introduction

In recent years, the field of medical imaging has undergone a transformative evolution, largely driven by the remarkable advancements in artificial intelligence (AI) and machine learning (ML) technologies [1–3]. The term *artificial intelligence* refers to the scientific domain focused on enabling machines to execute tasks typically reliant on human intelligence [4]. Within AI, conventional *machine learning* stands as a discipline wherein algorithms undergo training via established datasets, allowing machines to *learn*. These trained algorithms subsequently apply acquired knowledge to conduct diagnostic analyses on unfamiliar datasets [5]. *Deep learning*, another subset of AI, mirrors the neural structure of the human brain. Employing artificial neural networks comprising multiple *hidden layers*, this methodology tackles intricate problem-solving. The integration of these *hidden layers* empowers machines to continuously assimilate new information, enhancing their proficiency over time [6].

Among various medical imaging modalities, musculoskeletal (MSK) ultrasound (US) has gained increasing attention as a valuable diagnostic tool for assessing a wide range of disorders [7]. MSK US, with its non-invasive, radiation-free, and real-time imaging capabilities, is a valid solution for diagnosing and monitoring conditions such as tendon injuries, ligament tears, arthritis, and soft tissue abnormalities [8–10]. The application of AI in MSK US addresses some of the inherent challenges associated with conventional US imaging, including operator-dependent variability, image interpretation subjectivity, and time-consuming data analysis [11]. By leveraging AI algorithms, it is possible to automate the detection and characterization of MSK abnormalities, reducing the potential for human error and facilitating faster and more accurate diagnoses. Moreover, AI can aid in improving the standardization of image acquisition protocols and optimizing the overall workflow in MSK US examinations [12, 13].

To date, preliminary AI studies employing conventional ML or DL approaches have been applied to MSK US to improve diagnosis and outcome [14–16]. One issue is the validation of both conventional ML and DL approaches, which is crucial to ensure their generalizability [17]. The purpose of this systematic review was to assess the current state of research and development regarding AI integration in MSK US. We systematically reviewed and synthesized findings from a wide range of studies to evaluate the methodology, with emphasis on AI algorithm categories and validation strategies. We also discussed potential challenges, limitations, and future prospects of AI in MSK US, aiming to create awareness of important key topics when designing and executing future research related to AI in MSK US. Finally, this systematic review seeks to contribute to the growing body of evidence supporting the use of AI in MSK US, ultimately improving patient care, enhancing diagnostic capabilities, and advancing the field of MSK medicine.

## Material and methods

### Literature search

Local ethics committee approval was not needed because of the nature of the study, which was a systematic review.

An electronic literature search was conducted on the PubMed and Medline databases for articles published up to January 19, 2024. The search query was performed using the following keywords and their expansions: (“MSK” OR “musculoskeletal”) AND (“machine learning” OR “machine learning-based” OR “learning” OR “artificial intelligence” OR “artificial intelligence-based” OR “deep learning” OR “deep” OR “neural network”) AND (“ultrasound” OR “US”).

Studies were first screened by title and abstract, and then, the full text of eligible studies was retrieved for further review. The references of identified publications were checked for additional publications to include. The literature search and study selection were performed by one reviewer (blinded for review) and double-checked by a second reviewer (blinded for review). The Preferred Reporting Items for Systematic reviews and Meta-Analyses (PRISMA) guidelines [18] were followed.

### Inclusion and exclusion criteria

The inclusion criteria were (i) the use of AI in MSK US; (ii) involvement of human participants; (iii) English language; (iv) statement that approval from the local ethics committee and informed consent from each patient or a waiver for it was obtained.

The exclusion criteria were (i) studies reporting insufficient data for outcomes (e.g., details on AI algorithm and/or validation strategies not described); (ii) case reports and series, narrative reviews, guidelines, consensus statements, editorials, letters, comments, or conference abstracts.

### Data extraction

Data were extracted to a spreadsheet with a drop-down list for each item, grouped into three main categories, namely baseline study characteristics; AI algorithm categories; and validation strategies. Items regarding baseline study characteristics included first author’s last name, year of publication, study aim, evaluated structure, study design, sample size, and reference standard. Those concerning the AI algorithm included the use of conventional ML or DL-based algorithms. Data regarding validation strategies included the use of cross-validation techniques, clinical validation performed on a separate internal test dataset, and clinical validation performed on an external or independent test dataset.

## Results

### Baseline study characteristics

**Fig. 1:**
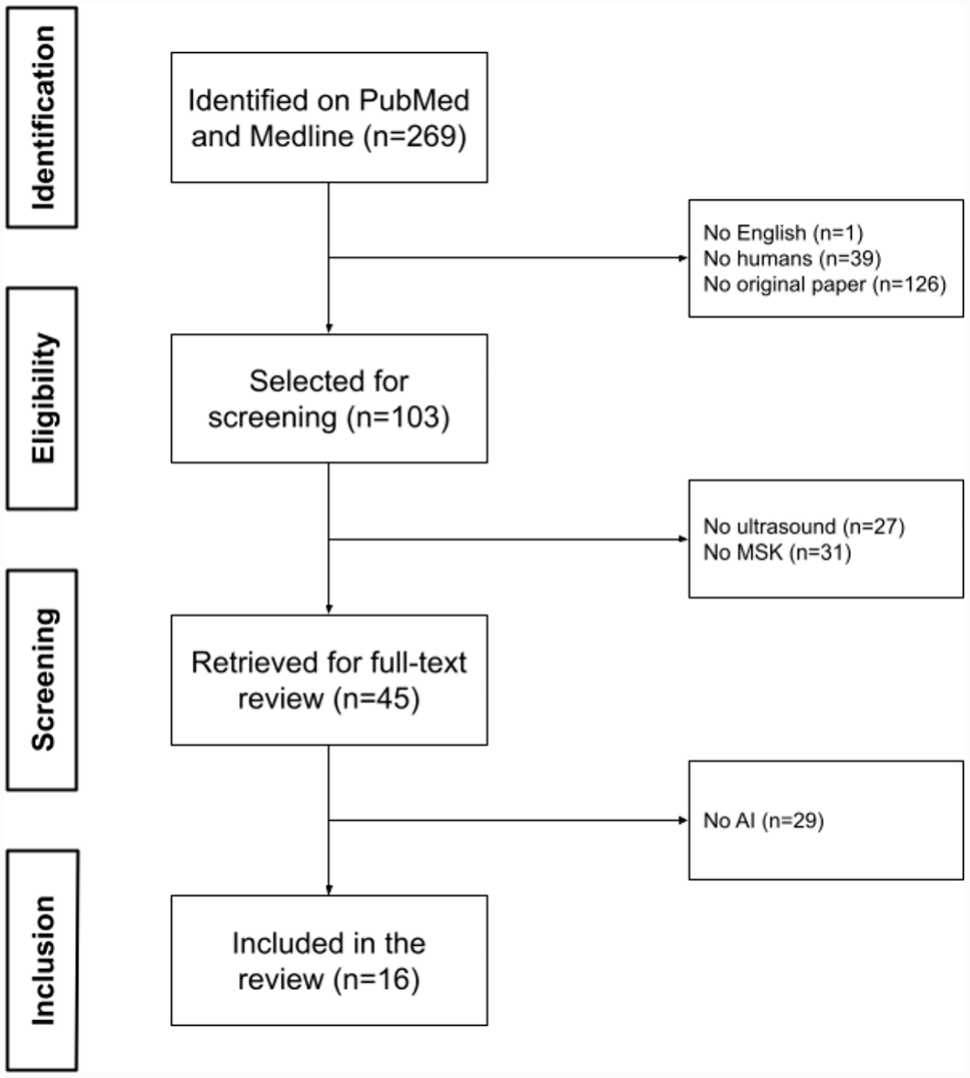
PRISMA (Preferred Reporting Items for Systematic reviews and Meta-Analyses) flowchart of systematic identification, screening, eligibility and inclusion information from retrieved studies. *MSK* indicates musculoskeletal, *AI* artificial intelligence

A flowchart illustrating the literature search process is presented in Fig. 1. After screening 269 papers and applying the eligibility criteria, 16 studies were included in this systematic review. Table 1 details the baseline study characteristics of the included studies.

**Table 1:** Baseline study characteristics of the papers dealing with artificial intelligence in musculoskeletal ultrasound included in the systematic review

First author	Year	Aim	Structure	Design	Number of patients (n)	Number of images (n)	Reference standard
Cheng [19]	2022	Diagnosis and grading of MCP synovitis	Hand	Prospective	446	446	Expert opinion/consensus
Chiu [20]	2022	Diagnosis of supraspinatus calcific tendinopathy	Shoulder	Retrospective	N/A	2462	Expert opinion/consensus
Cronin [21]	2020	Generation of synthetic US images	Lower leg	Prospective	52	100	Manual annotations
Cunningham [22]	2020	Estimation of absolute states of skeletal muscle	Lower leg	Prospective	32	403′023	EMG, ankle joint angle, ankle joint moment
Di Cosmo [23]/Smerilli [24]^a^	2022	Segmentation and CSA measurement of median nerve	Wrist	Prospective	103	246	Manual annotations
Droppelmann [25]	2022	Assessment of lateral elbow tendinopathy	Elbow	Retrospective	2917	30′007	Expert opinion/consensus
Du Toit [26]	2022	Segmentation of femoral articular cartilage	Knee	Prospective	25	25	Manual annotations
Lee [27]	2021	Assessment of developmental dysplasia of the hip	Hip	Retrospective	168	1243	Manual annotations
Lin [28]	2020	Diagnosis and grading of bicipital peritendinous effusion	Shoulder	Retrospective	3801	3801	Expert opinion/consensus
Loram [29]	2020	Analysis of neck muscle boundaries in cervical dystonia	Neck	Prospective	61	3272	Manual annotations
Lyu [30]	2023	Influence of ROI delineation methods on carpal tunnel syndrome diagnosis	Wrist	Retrospective	151	N/A	Nerve conduction
Marzola [31]	2021	Muscle segmentation for neuromuscular disease assessment	Upper arm, lower leg	Retrospective	1283	3917	Manual annotations
Rosa [32]	2021	Obtain muscle fascicle lengths in real-time	Lower leg	Prospective	6	8400	UltraTrack (automated muscle fascicle tracking software)
Saleh [33]	2021	Measurement of abdominal muscle thickness	Abdominal wall	Prospective	56	400	Manual annotations
Tiulpin [34]	2022	Prediction of total knee replacement	Knee	Prospective	557	N/A	Clinical registry
Yu [35]	2023	Differential diagnosis of pain rehabilitation of scapulohumeral periarthritis	Shoulder	Prospective	165	N/A	N/A

*MCP* indicates metacarpophalangeal, *US* ultrasound, *EMG* electromyography, *CSA* cross-sectional area, *ROI* region of interest

^a^Same study published in a medical journal (Di Cosmo et al. [23]) and in a scientific journal (Smerilli et al. [24])

All studies were published between 2020 and 2023. Two (12%) out of 16 investigations were published in 2023, six (38%) in 2022, four (25%) in 2021, and four (25%) in 2020. The design was prospective in 10 (62%) studies and retrospective in the remaining six (38%) studies. The median sample size was 151 patients (range 6 – 3,801).

The research was focused on diagnosis or grading of pathologies in seven (44%) studies and segmentation of structures in four (25%) studies. Less frequently, research was aimed at the prediction of total knee replacement outcome [34], generation of synthetic US images [21], estimation of absolute states of skeletal muscle [22], assessment of muscle fascicle lengths [32], and measurement of muscle thickness [33], as detailed in Table 1. Regarding the anatomic structures investigated, eight (50%) papers focused on the upper extremity, with the shoulder being the most frequently investigated structure (*n* = 3, 19%). Seven (44%) papers focused on the lower extremity, with the lower leg being the most frequently investigated structure (*n* = 4, 25%).

The most frequently employed reference standard was manual annotation (*n* = 7, 44%), followed by expert opinion/consensus (*n* = 4, 25%). In the remaining studies, electromyography [22], nerve conduction studies [30], an automated muscle fascicle tracking software [32], and clinical follow-up information [34] were used as reference standards. In one study [35], the reference standard was not specified.

### AI algorithms and validation strategies

A total of 11 (69%) studies used DL-based algorithms, three (19%) studies employed conventional ML-based algorithms, and two (12%) studies employed both DL and conventional ML algorithms, as detailed in Table 2.

**Table 2:** Details on artificial intelligence algorithm categories and validation strategies used in the papers included in the systematic review

First author	Algorithm category	Cross-validation	Internal clinical validation	External clinical validation
Cheng [19]	Deep learning	N/A	Yes	No
Chiu [20]	Deep learning	N/A	Yes	No
Cronin [21]	Deep learning	N/A	No	No
Cunningham [22]	Deep learning	K-fold cross-validation	No	No
Di Cosmo [23]/Smerilli [24] ^a^	Deep learning	K-fold cross-validation	Yes	No
Droppelmann [25]	Conventional machine learning	K-fold cross-validation	Yes	No
Du Toit [26]	Deep learning	N/A	Yes	No
Lee [27]	Deep learning	N/A	Yes	No
Lin [28]	Deep learning	N/A	Yes	No
Loram [29]	Conventional machine learning, deep learning	Leave-one-out cross-validation	No	No
Lyu [30]	Conventional machine learning, deep learning	N/A	Yes	No
Marzola [31]	Deep learning	N/A	Yes	No
Rosa [32]	Conventional machine learning	N/A	No	No
Saleh [33]	Deep learning	K-fold cross-validation	No	No
Tiulpin [34]	Conventional machine learning	Leave-one-out cross-validation	No	No
Yu [35]	Deep learning	N/A	No	No

^a^Same study published in a medical journal (Di Cosmo et al. [23]) and in a scientific journal (Smerilli et al. [24])

Regarding validation strategies, a total of six (38%) studies provided details on cross-validation techniques with K-fold cross-validation being the most frequently used (*n* = 4, 25%). Two (13%) studies used the leave-one-out cross-validation technique. A clinical validation was reported in nine (56%) papers. In all cases, the clinical validation was performed on a separate set of data from the primary institution, i.e., internal test dataset. No cases of external or independent validation were reported in the papers included in this systematic review. Details on cross-validation and clinical validation strategies employed in the included studies are provided in Table 2.

## Discussion

This systematic review focused on the use of AI in MSK US with emphasis on categories of AI algorithms and validation strategies. Most included studies employed DL-based algorithms, either alone or combined with conventional ML approaches. Clinical validation with internal test datasets was frequently used. However, no cases of external validation were reported.

AI has gained increasing attention in medicine and particularly also in radiology over the past few years [1]. The same is true for MSK imaging as a radiological sub-specialty. The number of papers that focused on AI in MSK US has increased over the years, and half of those included in this review have been published since 2022. Compared to the total literature published on AI in MSK imaging, the papers focusing on US imaging are a minority, however. The authors believe that this might be explained by several reasons: (i) US imaging is less popular than alternative imaging methods for diagnosing MSK pathologies in many centers and (ii) US imaging is more operator dependent than other modalities, which makes it more difficult to standardize and successfully train AI models for the purpose of diagnosis and/or grading of pathologies [11].

Fields of application of AI in MSK US are diverse. Most studies in this systematic review focused on clinical questions related to diagnosis and/or grading of MSK pathologies. They used a prospective study design in a majority of cases which offers advantages in controlling data gathering and matching patient and/or imaging characteristics. Retrospective study designs, which were used to a lesser extent, allowed inclusion of a larger number of patients with imaging data previously acquired. Public databases were not used in the papers included in this review and should be considered in future research studies to validate AI approaches against independent data.

The AI algorithms most frequently encountered were based on DL. Compared with conventional ML, DL can automatically filter features to improve recognition performance based on multi-layer models [36]. However, a large number of labeled training samples are required in order to achieve excellent learning performance [37]. This requirement can be difficult to meet in US imaging where expert annotation is time-consuming and datasets are often limited with regard to the number of cases.

Validation of AI performance is important. When dealing with limited datasets, resampling strategies such as cross-validation prove beneficial. They aim to curb over fitting and improve the accuracy of the model's performance on new data [38]. Among the studies reviewed, K-fold cross-validation was the most frequently utilized technique for this purpose. At the same time, a clinical validation against a separate set of data is desirable to test the AI model and ensure its applicability on unseen cases. Clinical validation with internal test datasets was performed in the majority of papers. However, none of the studies conducted clinical validation using entirely separate datasets from different institutions. Hence, for future studies, it would be crucial to expand beyond a single institution and incorporate external testing of the model with substantial and independent datasets. This approach would greatly enhance the robustness and reliability of the findings.

### Limitations

This study represents a systematic review of the literature. Due to the limited number of papers dealing with AI in MSK US published over the past few years and their heterogeneity with regard to different categories analyzed and metrics employed, it was not possible to perform a meta-analysis with more solid statistical tests. Furthermore, the review did not include a formal evaluation of the quality of each study that was included. Our emphasis was on presenting methodological data that serve as quality indicators on their own.

## Conclusion

AI is a topic of increasing interest in MSK US research reflected by the growing number of publications each year. Regarding the methodology of such studies, attention should be paid to the use of accurate reproducibility and validation strategies in order to assure high-quality algorithms and outcomes.

## Abbreviations

AI: Artificial intelligence
DL: Deep learning
ML: Machine learning
MSK: Musculoskeletal
US: Ultrasound
